# Identification of the Biosynthetic Gene Cluster for the Organoarsenical Antibiotic Arsinothricin

**DOI:** 10.1128/spectrum.00502-21

**Published:** 2021-08-11

**Authors:** Adriana E. Galván, Ngozi P. Paul, Jian Chen, Kunie Yoshinaga-Sakurai, Sagar M. Utturkar, Barry P. Rosen, Masafumi Yoshinaga

**Affiliations:** a Department of Cellular Biology and Pharmacology, Florida International University, Herbert Wertheim College of Medicine, Miami, Florida, USA; b Purdue University Center for Cancer Research, Purdue University, West Lafayette, Indiana, USA; Shenzhen Bay Laboratory

**Keywords:** arsinothricin, organoarsenical antibiotic, biosynthetic gene cluster, *Burkholderia gladioli* GSRB05

## Abstract

The soil bacterium Burkholderia gladioli GSRB05 produces the natural compound arsinothricin [2-amino-4-(hydroxymethylarsinoyl) butanoate] (AST), which has been demonstrated to be a broad-spectrum antibiotic. To identify the genes responsible for AST biosynthesis, a draft genome sequence of *B. gladioli* GSRB05 was constructed. Three genes, *arsQML*, in an arsenic resistance operon were found to be a biosynthetic gene cluster responsible for synthesis of AST and its precursor, hydroxyarsinothricin [2-amino-4-(dihydroxyarsinoyl) butanoate] (AST-OH). The *arsL* gene product is a noncanonical radical *S*-adenosylmethionine (SAM) enzyme that is predicted to transfer the 3-amino-3-carboxypropyl (ACP) group from SAM to the arsenic atom in inorganic arsenite, forming AST-OH, which is methylated by the *arsM* gene product, a SAM methyltransferase, to produce AST. Finally, the *arsQ* gene product is an efflux permease that extrudes AST from the cells, a common final step in antibiotic-producing bacteria. Elucidation of the biosynthetic gene cluster for this novel arsenic-containing antibiotic adds an important new tool for continuation of the antibiotic era.

**IMPORTANCE** Antimicrobial resistance is an emerging global public health crisis, calling for urgent development of novel potent antibiotics. We propose that arsinothricin and related arsenic-containing compounds may be the progenitors of a new class of antibiotics to extend our antibiotic era. Here, we report identification of the biosynthetic gene cluster for arsinothricin and demonstrate that only three genes, two of which are novel, are required for the biosynthesis and transport of arsinothricin, in contrast to the phosphonate counterpart, phosphinothricin, which requires over 20 genes. Our discoveries will provide insight for the development of more effective organoarsenical antibiotics and illustrate the previously unknown complexity of the arsenic biogeochemical cycle, as well as bring new perspective to environmental arsenic biochemistry.

## INTRODUCTION

Arsenic is one of the most ubiquitous environmental toxic substances. Bacteria have taken advantage of its prevalence to evolve mechanisms that give them competitive advantages over bacterial competitors. These include pathways for arsenic respiration for energy generation (1–3), methylation of inorganic arsenic to increase its potency as an antimicrobial (4, 5), and even incorporation into arsenolipids for sparing phosphate under nutrient-limiting conditions (6, 7). A newly recognized adaptation is the synthesis of a novel arsenic-containing antibiotic.

Antibiotic resistance is a global health challenge, and new antibiotics are urgently needed. Recently the rice rhizosphere bacterium Burkholderia gladioli GSRB05 was shown to produce the broad-spectrum arsenic-containing antibiotic arsinothricin (2-amino-4-hydroxymethylarsinoyl) butanoate (AST) (8). AST effectively inhibits growth of the World Health Organization (WHO) priority pathogens such as carbapenem-resistant Enterobacter cloacae (CRE) but has low cytotoxicity in human monocytes (9). It also inhibits growth of Mycobacterium bovis BCG, which causes tuberculosis (TB) in animals and humans and is closely related to Mycobacterium tuberculosis, the major causative agent of human TB. TB is classified by the WHO as a global health emergency (10), and the WHO has called for the development of new and innovative antibiotics against M. tuberculosis (https://www.who.int/activities/tackling-the-drug-resistant-tb-crisis). AST is a pentavalent organoarsenical and a nonproteinogenic amino acid analog of glutamate. It inhibits glutamine synthetase (GS), most likely because of its chemical similarity to the acyl-phosphate intermediate γ-glutamyl phosphate, in the GS catalytic cycle (9). GS is essential for nitrogen metabolism in M. tuberculosis, and inhibitors of M. tuberculosis GS are actively sought after as drugs against TB (11).

*B. gladioli* GSRB05 is the only known AST producer that has been shown to produce both pentavalent hydroxyarsinothricin [2-amino-4-(dihydroxyarsinoyl) butanoate] (AST-OH) and AST from As(III), with a possible precursor-product relationship (8). Here, we identified the biosynthetic gene cluster (BGC) for AST production from the genome of *B. gladioli* GSRB05. Three genes, *arsQ*, *arsM*, and *arsL*, are organized in an arsenic resistance (*ars*) operon. By way of comparison, the phosphonate analog of AST is the *Streptomyces* antibiotic phosphinothricin, which is used commercially as the herbicide glufosinate and has a very complicated biosynthetic pathway (12). The BGC from Streptomyces viridochromogenes consists of 24 genes (accession no. X65195) (13), so a three-gene BGC for arsinothricin production is surprisingly small.

Identification of the AST BGC makes substantial contributions in two important areas—treatment of infectious diseases and radical *S*-adenosylmethionine (SAM) chemistry. First, the BGC is an uncomplicated pathway with only three steps required for the synthesis of AST. This arsenic-containing compound may be the founding member of a new class of antibiotics, adding to our arsenal of weapons against multidrug-resistant pathogens. Second, the radical SAM enzyme BgArsL (i.e., *B. gladioli* ArsL) catalyzes the key enzymatic reaction in AST biosynthesis. Radical SAM enzymes form the largest enzyme superfamily (14). Most members catalyze the transfer of a 5′-deoxyadenosyl radical to the substrate or function as methyltransferases using a methylene fragment from SAM (15). In contrast, BgArsL is a noncanonical radical SAM enzyme that transfers the 3-amino-3-carboxypropyl moiety to As(III), forming AST-OH, a unique radical SAM reaction.

## RESULTS

### Identification of the AST biosynthetic gene cluster.

**FIG 1:**
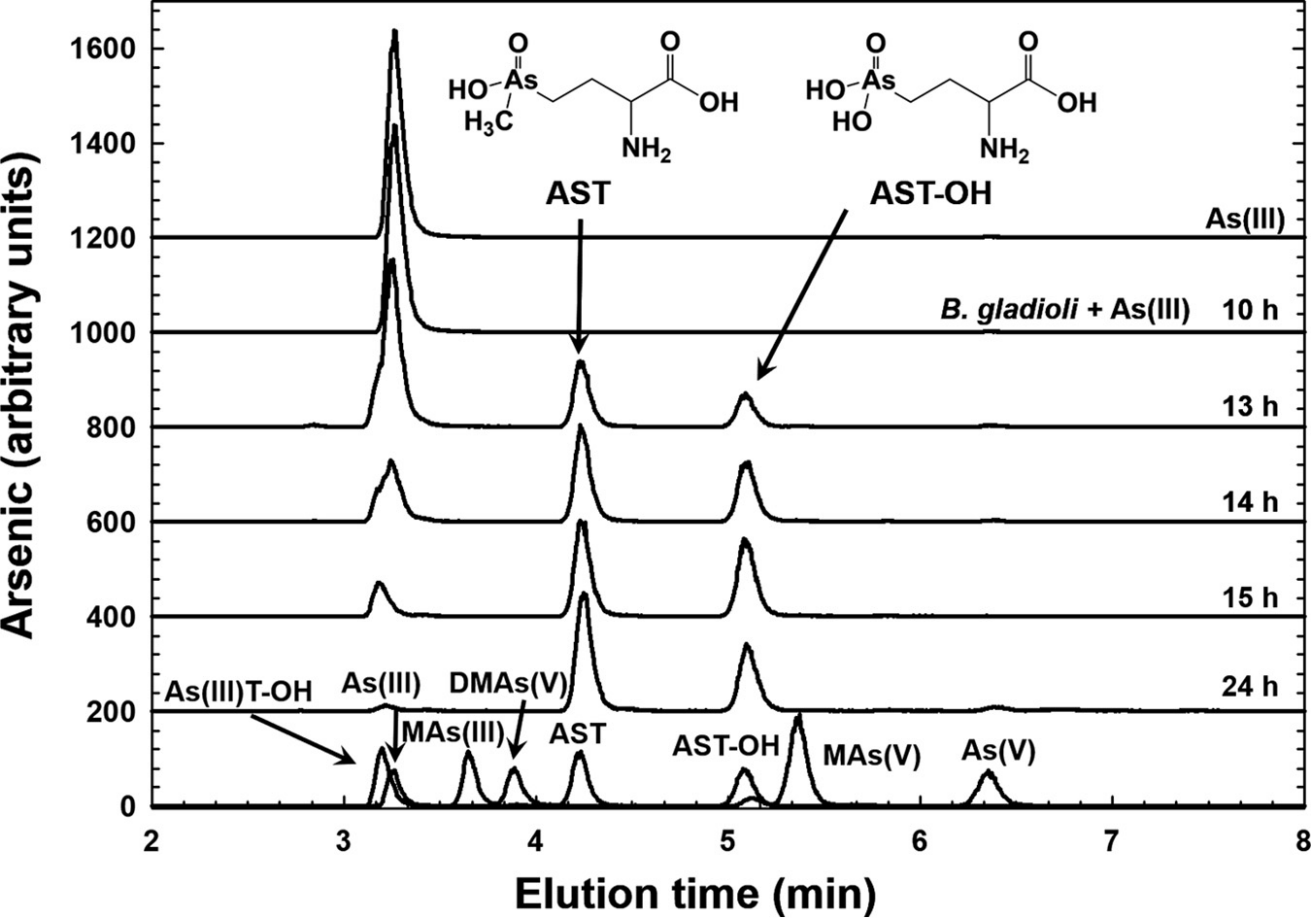
Time course of AST biosynthesis by *B. gladioli* GSRB05. Production of AST-OH and AST by cells of *B. gladioli* GSRB05 was assayed over a 24-h period. Cells were grown in LB medium until late log phase and transferred to ST 10^−1^ for up to 24 h in the presence of 3 μM As(III). Samples were speciated by C_18_ reverse-phase HPLC, and arsenic was determined by ICP-MS at the indicated times. After a lag period of approximately 10 h, AST-OH and AST synthesis was observed. During the remaining time, the amount of AST increased, and As(III) decreased correspondingly. Production of AST-OH leveled off after approximately 14 to 15 h, indicating that it is an intermediate in the pathway of AST biosynthesis from As(III). (Bottom line) Standards of arsenic compounds. As(III)T-OH, trivalent hydroxyarsinothricin; As(III), arsenite; MAs(III), methylarsenite; DMAs(V), dimethylarsenate; AST, arsinothricin; AST-OH, hydroxyarsinothricin; MAs(V), methylarsenate; As(V), arsenate.

In this study, a time course of AST biosynthesis by *B. gladioli* GSRB05 was conducted in cells grown in ST 10^−1^ medium (Fig. 1). After a lag period of approximately 10 h, both trivalent and pentavalent AST-OH and pentavalent AST were produced. After approximately 15 h, all of the As(III) was consumed, the trivalent AST-OH peak decreased, and both the AST and the pentavalent AST-OH peaks increased correspondingly. We interpret this result as As(III) conversion into trivalent AST-OH in the first step, which is then either methylated to AST in a second step or oxidized to pentavalent AST-OH. After 24 h, both the pentavalent and trivalent AST-OH peaks decreased, and the AST peak increased reciprocally, suggesting that the strain is able to reduce pentavalent AST-OH to the trivalent form to produce more AST. It is not clear if the product is trivalent or pentavalent AST because in our hands trivalent AST oxidizes in air too rapidly to be isolated. Trivalent AST-OH also oxidizes in air, but less rapidly than AST.

**FIG 2:**
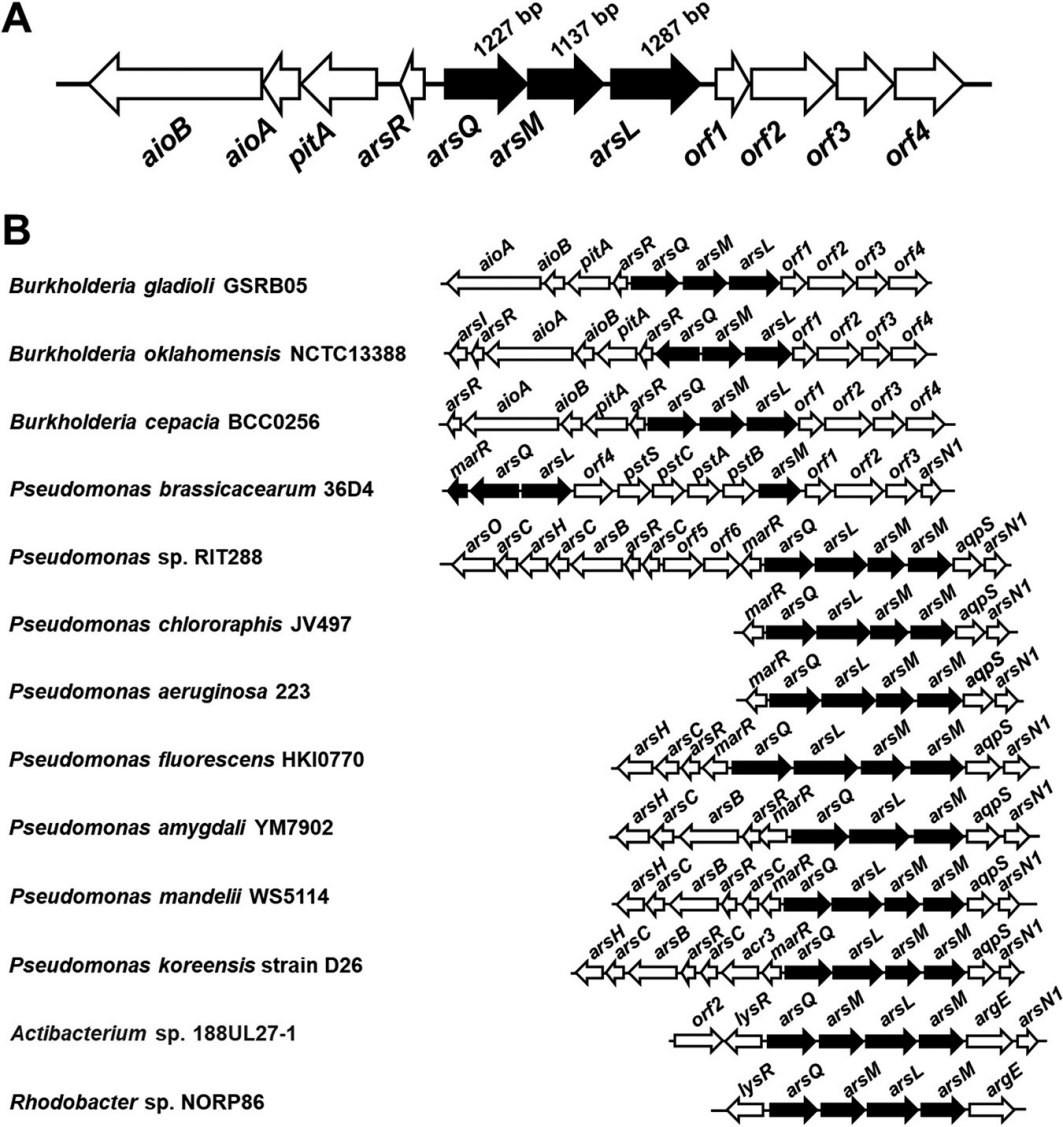
The *B. gladioli* GSRB05 AST biosynthetic gene cluster. (A) The *B. gladioli* GSRB05 genome (accession no. JAGSIB000000000) contains a cluster of *ars* genes, including *arsQML*. (B) Phylogenetic distribution of the genes of the AST BGC. GenBank accession numbers are given in Materials and Methods.

To identify the AST BGC knowing that methylation is involved, we made the assumption that a *B. gladioli* GSRB05 enzyme would be related to other known methylating enzymes. Arsenic methylation catalyzed by the ArsM As(III) *S*-adenosylmethionine methyltransferase is a common reaction in arsenic metabolism (16). Bacterial and algal ArsM enzymes methylate inorganic As(III) up to three times to produce methylarsenite [MAs(III)], dimethylarsenite [DMAs(III)], and trimethylarsenite [TMAs(III)]. These are rapidly oxidized nonenzymatically in air to the pentavalent species, which are neither substrates nor products of the enzyme-catalyzed reaction. We predicted that a *B. gladioli* GSRB05 ArsM ortholog is involved in the methylation of trivalent AST-OH and that its gene would be in the AST BGC. For that reason, a draft sequence of the *B. gladioli* GSRB05 genome was constructed (see Fig. S2 in the supplemental material). As anticipated, an *arsM* sequence was identified next to a cluster of genes related to arsenic metabolism but divergently oriented (Fig. 2A). These genes located on the opposite DNA strand are *arsR* (17), *pitA* (18), *aioAB* (2), encoding a putative ArsR As(III)-responsive transcriptional repressor, PitA, a low-affinity inorganic phosphate/arsenate transporter, and the AioAB arsenite oxidase that oxidizes As(III) to As(V), respectively. The predicted products of the genes upstream and downstream of *arsM* were searched against the basic local alignment search tool (BLAST; http://blast.ncbi.nlm.nih.gov) using the BLASTP program. The gene immediately upstream of *arsM* is termed *arsQ* and encodes a putative membrane transporter annotated as related to the GntP family of gluconate permeases (19). The gene immediately downstream of *arsM* encodes a putative radical SAM protein and is termed *arsL.* The *arsQML* cluster is found in the genomes of other proteobacterial genomes but is not widespread in the bacterial kingdom (Fig. 2B). Their association suggests that these three genes have a related function. The next four genes were termed *orf1–4* and are annotated as encoding two class I SAM-dependent methyltransferases, a cytochrome P450-like protein, and an α/β-hydrolase. The four genes are not adjacent to *arsQML* in other bacterial genomes, and as described below, these four genes appear to be unrelated to AST biosynthesis.

**FIG 3:**
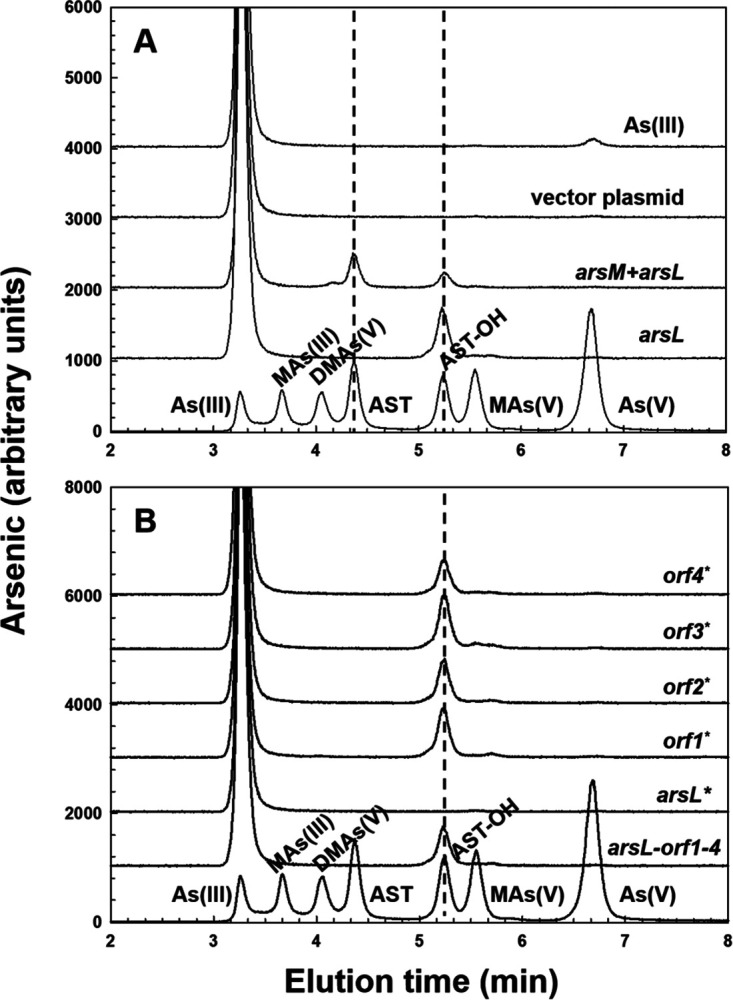
AST-OH and AST biosynthesis in cells bearing *arsML–orf1–4*. As(III) biotransformation was assayed with selected genes from the AST BGC from *B. gladioli* GSRB05. (A) Cultures of E. coli Top10 expressing *arsML–orf1–4* and/or *arsL–orf1–4* in plasmid pUC118. From the bottom the lanes are arsenic standards, cells with 1 μM As(III) bearing pUC118*arsL–orf1–4*, pUC118*arsML–orf1–4*, vector plasmid pUC118, and medium with only 1 μM As(III). (B) Stop codons (*) were individually introduced into the genes in plasmid pUC118*arsL–orf1–4*. From the bottom, the lanes are arsenic standards, cells expressing wild type *arsL–orf1–4* genes, and stop codons in each gene. Cultures were grown in LB medium to late log phase, transferred to ST 10^−1^ medium with 1 μM As(III), and incubated for 36 h. Arsenic-containing species were determined by HPLC-ICP-MS.

The *B. gladioli arsM* gene product (BgArsM) is a 378-amino-acid-residue protein with a predicted mass of 41.71 kDa (accession no. MBW5287222). Most ArsM and related animal AS3MT enzymes have four conserved cysteine residues that are required for catalysis (20, 21). Multiple-sequence alignment of BgArsM and orthologs shows that BgArsM has four cysteine residues at positions 30, 54, 181, and 233 (see Fig. S3 in the supplemental material). The N terminus of BgArsM does not align well with those of other ArsMs, but Cys30 is in approximately the position expected for the first conserved cysteine, and the other three align well with the remaining three conserved cysteine residues. The *arsL* gene product (BgArsL) is a 428-amino-acid-residue protein with a predicted mass of 47.5 kDa (accession no. MBW5287221). Multiple-sequence alignment with other putative ArsL orthologs shows that BgArsL has three conserved cysteine residues at positions 194, 198, and 201 (see Fig. S4 in the supplemental material). It is annotated as a radical SAM enzyme, with these three cysteine residues forming a CX_3_CX_2_C motif that is found in more than 90% of members of the radical SAM superfamily. In the cyanobacterium *Synechocystis* sp. strain PCC 6803, a radical SAM enzyme, SsArsS, has been shown to function with SsArsM to catalyze the initial steps in arsenosugar biosynthesis (22). It was therefore reasonable to consider that BgArsM and BgArsL might function together in AST biosynthesis. The *arsQML* genes plus the four downstream *orf* genes were cloned and transformed into Escherichia coli Top10. However, the transformants grew poorly. Considering that the heterologous expression of membrane proteins, in general, might be toxic for the host cells, *arsQ* was eliminated from the construct, leaving *arsML* and the four *orf* genes. Cells of E. coli Top10 with the remaining six genes grew well, indicating that *arsQ* was responsible for growth retardation. With the addition of 1 μM As(III), cells expressing the six genes produced AST and smaller amounts of AST-OH (Fig. 3A). Cells expressing an *arsL–orf1–4* construct lacking *arsM* produced only AST-OH and not AST. These results indicate that one or more of the five genes downstream of *arsM* are involved in AST-OH biosynthesis, followed by methylation to AST by BgArsM.

### BgArsM and BgArsL are both required for AST biosynthesis.

**FIG 4:**
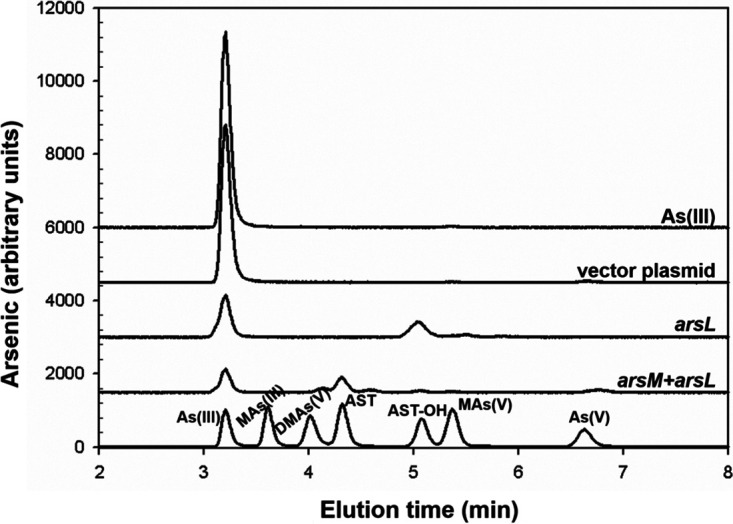
The *arsL* and *arsM* gene products are sufficient to catalyze sequential steps in the biosynthesis of AST. Production of AST-OH or AST was assayed in cultures of E. coli BL21 with 1 μM As(III), as described in the legend to Fig. 3. From the bottom, the lanes are arsenic standards, cells bearing pETDuet-1*arsLM*, pETDuet-1*arsL*, vector plasmid pETDuet-1, and medium with only 1 μM As(III).

To determine which gene or genes are required for AST-OH production, a mutation producing a stop codon was introduced into *arsL* and each of the other four genes in the *arsL–orf1–4* construct. Cells expressing each mutant were incubated with As(III), and the culture medium was analyzed by high-performance liquid chromatography-inductively coupled plasma mass spectrometry (HPLC-ICP-MS). Stop codons in the downstream *orf1–4* genes had no effect, and only the strain with the point mutation in the *arsL* gene lost the ability to produce AST-OH (Fig. 3B). Thus, only *arsL* is required for AST-OH production. These results strongly suggest that the biosynthetic pathway of AST is composed of only two reactions catalyzed by BgArsM and BgArsL. To confirm this hypothesis, we expressed *arsL* alone and the *arsML* genes together in the pETDuet-1 system (Fig. 4). Cells of E. coli BL21 expressing *arsL* alone produced AST-OH but not AST. In contrast, AST production was observed when *arsL* and *arsM* were coexpressed.

### The *arsQML* genes comprise an As(III)-inducible *ars* operon.

**FIG 5:**
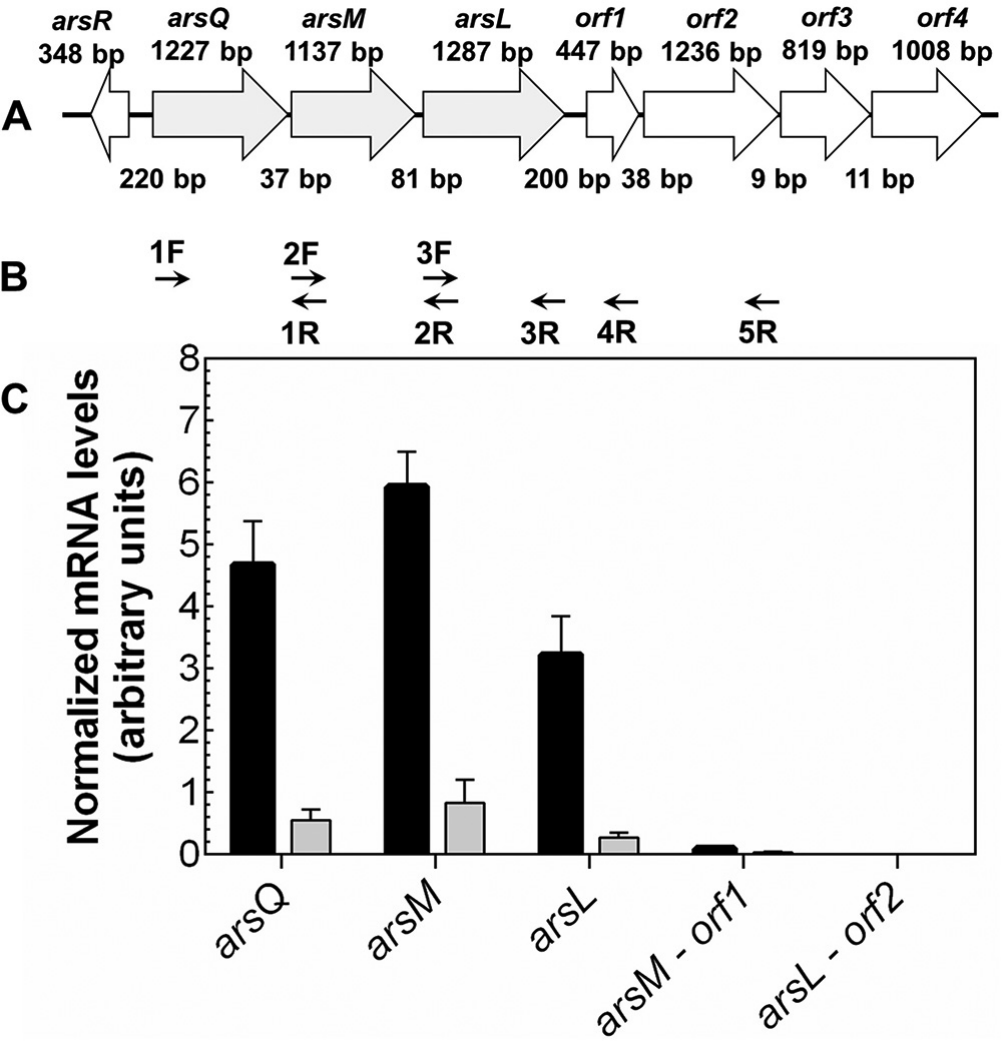
The BGC is the *arsQML* operon. (A) The BGC and upstream and downstream genes are illustrated, with the size of each gene and intergenic regions given in nucleotide base pairs. (B) The indicated forward (F) and reverse (R) primers were used to amplify transcriptional products by RT-PCR. (C) Analysis of transcription of the *arsQ* (primers 1F-1R), *arsM* (primers 2F-2R), or *arsL* (primers 3F-3R) genes individually and cotranscription of *arsM–orf1* (primers 2F-4R) and *arsL–orf2* (primers 3F-5R) genes. *B. gladioli* GSRB05 was grown in LB medium until late log phase and then transferred to ST 10^−1^ medium. Cultures were incubated for 13 h in the presence (black bars) or absence (shaded bars) of 3 μM As(III). cDNA was synthesized by RT-PCR from total RNA obtained from cultures of *B. gladioli* GSRB05. Data are the mean ± SE (*n* = 4).

To examine whether the *arsQ*, *arsL*, and *arsM* genes comprise an As(III)-responsive *ars* operon, total RNA was obtained from cells of *B. gladioli* GSRB05 with or without As(III), and cDNA was subsequently synthesized by reverse transcription-PCR (RT-PCR). From the amount of RNA detected in quantitative real-time PCR (RT-qPCR) analysis, expression of *arsQ*, *arsM*, and *arsL* increased approximately 8.5-, 7.2-, and 11.9-fold, respectively, following induction with 3 μM As(III) compared with RNA from uninduced cells (Fig. 5). The results demonstrate that the *arsQML* genes comprise an *ars* operon. As(III) responsiveness suggests that the operon is controlled by an ArsR repressor, likely the product of the upstream *arsR* gene. When the reverse primers were designed to anneal a cDNA region including either *orf1* or *orf2*, almost no amplification was observed, indicating that the downstream four *orf* genes are not part of the *arsQML* operon.

### ArsQ is an AST efflux permease.

Antibiotic BGCs frequently have genes for efflux of the antibiotic. This serves dual purposes of removing the active antibiotic from the cytosol, thus protecting the producing organism, and exporting the compound into the medium, where it can exert its antibiotic action against other bacteria. The *arsQ* gene encodes a putative membrane protein of 408 amino acid residues with a predicted mass of 42.9 kDa (accession no. MBW5287223). ArsQ orthologs are found primarily in *Proteobacteria* and are annotated as gluconate permeases. AST and gluconate are similar in size and charge, so a reasonable inference is that the BgArsQ functions as an AST efflux permease. A multiple-sequence alignment with the 100 most closely related proteins shows variation in the N-terminal 90 residues, but the remaining 318 residues are highly conserved (see Fig. S5 in the supplemental material). The *arsQ* gene was cloned into vector pTrcHisA and transformed into the As(III)-hypersensitive strain E. coli AW3110 (23).

**FIG 6:**
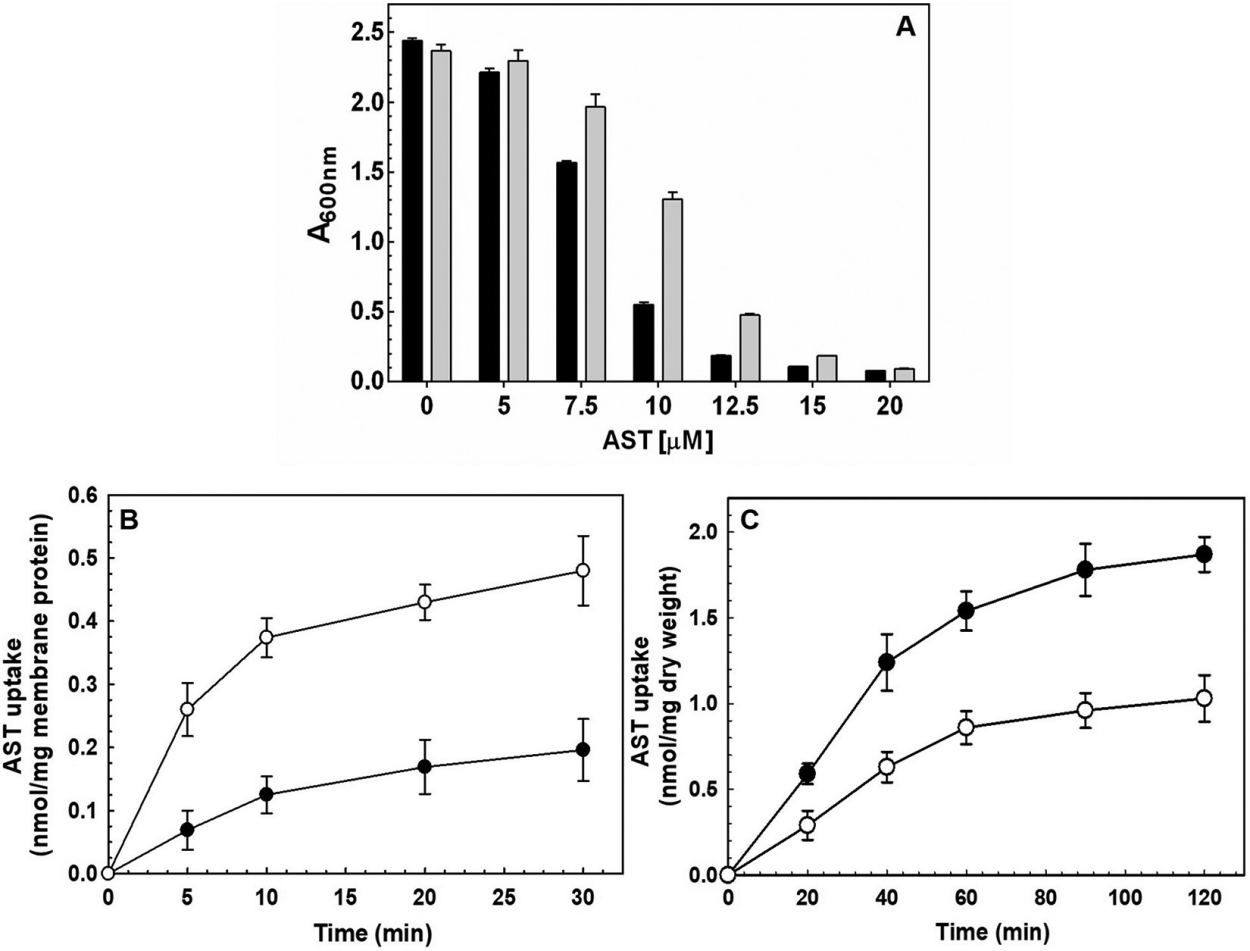
BgArsQ is an AST efflux permease. (A) Expression of *arsQ* confers resistance to AST. Overnight cultures of E. coli AW3110 bearing either pTrcHisA*arsQ* (shaded bars) or vector plasmid pTrcHisA (black bars) were diluted 100-fold into fresh M9 medium containing the indicated concentrations of AST. *A*_600_ was measured after 24 h of growth at 37°C. (B) Uptake of AST (40 μM) was assayed in cells of E. coli AW3110 bearing either pTrcHisA*arsQ* (●) or vector plasmid pTrcHisA (○). (C) Uptake of AST (20 μM) was assayed in everted vesicles prepared from cells of E. coli AW3110 expressing pTrcHisA*arsQ* (●) or vector plasmid pTrcHisA (○) with 5 mM NADH as an energy source, as described in Materials and Methods. Data are the mean ± SE (*n* = 3).

Addition of the inducer IPTG (isopropyl-β-d-1-thiogalactopyranoside) produced growth inhibition, so the cells were grown without inducer, and the effect of leaky expression of *arsQ* on AST inhibition was assayed (Fig. 6A). Cells expressing *arsQ* were clearly more resistant to AST than those with vector only. The resistance was not dramatic—probably due to low levels of expression of BgArsQ. Next, the effect of *arsQ* expression on accumulation of AST was examined. Cells expressing BgArsQ accumulated significantly less AST than the control (Fig. 6B). Uptake into everted vesicles reflects efflux from cells (24, 25), so everted membrane vesicles were prepared from those cells, and energy-driven uptake of AST into those vesicles was assayed. Cells expressing BgAST took up significantly more AST than the control (Fig. 6C). These results demonstrate that expression of *arsQ* increases resistance by active efflux of AST.

## DISCUSSION

**FIG 7:**
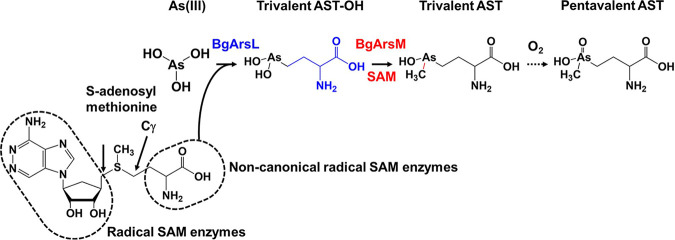
Proposed pathway of AST biosynthesis. AST biosynthesis by *B. gladioli* GSRB05 is composed of two steps. In the first step, the noncanonical radical SAM enzyme BgArsL cleaves the Cγ bond of SAM, forming a 3-amino-3-carboxypropyl (ACP) radical that creates a C-As bond with As(III), producing trivalent AST-OH. This is different from most radical SAM enzymes that form 5′-deoxyadenosyl radical by reductive cleavage of SAM at the 5′ position of the adenosine moiety. In the second step, BgArsM uses a second molecule of SAM to methylate trivalent AST-OH, generating trivalent AST, which spontaneously oxidizes nonenzymatically to the antibiotic pentavalent AST.

Identification of the biosynthetic gene cluster for arsinothricin is an essential step in elucidation of the antimicrobial action of this novel arsenic-containing antibiotic. With only three genes, the BGC is surprisingly small, especially compared with the BGC for the phosphonate counterpart, phosphinothricin, which includes 24 genes. The AST BGC has a rather narrow phylogenetic distribution. The *arsQML* gene cluster is found primarily in members of the *Proteobacteria* phylum, such as the classes *Alphaproteobacteria* (*Rhodobacter*), *Betaproteobacteria* (*Burkholderia*), and *Gammaproteobacteria* (Pseudomonas). Most BgArsQ orthologs are found in *Proteobacteria*, while orthologs of BgArsM and BgArsL are more widely distributed. However, it is not clear if those are involved in AST biosynthesis. Near other *arsL* genes are also genes for other putative permeases that are unrelated to ArsQ. It is not known if their substrates include AST, but alternate AST transporters would not be surprising. This is reminiscent of the existence of multiple permeases for inorganic and organic arsenicals, such as ArsB, Acr3, ArsP, ArsK, and ArsJ (26). We propose that AST is synthesized in a three-step pathway: (i) AST-OH synthesis by BgArsL, (ii) methylation of AST-OH to AST by BgArsM, and (iii) export of AST by BgArsQ (Fig. 7).

BgArsL can be predicted to be a radical SAM enzyme, a member of the largest enzyme superfamily. It has three cysteine residues (Cys194, Cys198, and Cys201) that are highly conserved in radical SAM enzymes and form a [4Fe-4S]^+^ cluster for reductive cleavage of SAM. Since BgArsL has only three conserved cysteine residues, it likely has a single [4Fe-4S]^+^ cluster. The majority of radical SAM enzymes produce a 5′-deoxyadenosyl radical that performs a wide variety of organic chemical biotransformations (15). AST-OH is an amino acid with arsenate replacing the γ-carboxyl group of glutamic acid, so it is reasonable to propose that BgArsL catalyzes addition of the 3-amino-3-carboxypropyl (ACP) group of SAM to As(III), forming trivalent AST-OH, as shown in Fig. 7. In this respect, BgArsL is a novel noncanonical radical SAM enzyme that forms an ACP radical rather than the more common 5′-deoxyadenosyl radical. This reaction would produce trivalent AST-OH. The only other noncanonical radical SAM enzyme that transfers ACP is the diphthamide biosynthetic enzyme Dph2, which adds ACP to a histidine residue in translation elongation factor 2 (27). Rather than catalyzing protein modification like Dph2, ArsL catalyzes the unique chemistry of C-As bond formation. Enzymes with [4Fe-4S]^+^ clusters are typically very oxygen sensitive, usually requiring anaerobic purification and/or reconstitution of the iron-sulfur cofactor (28). Future directions of study with BgArsL will be designed for the challenging characterization of the enzyme *in vitro*.

BgArsM is a typical As(III) SAM methyltransferase. ArsM substrates include inorganic arsenite, methylarsenicals, and aromatic arsenicals. A mechanistic feature of this group of arsenic methyltransferases is that both the substrates and products are trivalent, and the pentavalent species are produced nonenzymatically by oxidation, usually with atmospheric O_2_ (16). Cells of E. coli heterologously expressing the *arsL* gene transform As(III) into AST-OH, and cells expressing both *arsL* and *arsM* genes produce ATS. Thus, we propose that in the second step, trivalent AST-OH is methylated by BgArsM to form the reduced form of AST, which would rapidly oxidize, yielding the pentavalent antibiotic AST. Future tests of this proposal will involve purification of BgArsM for enzymatic assays and development of analytical methods to detect the trivalent form of AST.

Antibiotic producers frequently have transporters that transport the active antibiotic from the cells, both for self-protection and to inhibit growth of competitors (29). In the third and final step, AST is exported from the cells by the efflux permease BgArsQ. The results of transport assays with cells of E. coli heterologously expressing *arsQ* clearly demonstrate that BgArsQ reduces the intracellular concentration of AST, and in complementary assays with everted membrane vesicles, there is energy-dependent uptake of AST. It is not known whether BgArsQ substrates are trivalent, pentavalent, or both, whether AST-OH and AST are both substrates, or even if gluconate is a substrate, due to apparent toxicity caused by *arsQ* expression. Future experiments will be designed to produce sufficient BgArsQ for biochemical characterization.

## MATERIALS AND METHODS

### Strains, plasmid, media, and growth conditions.

Strains and plasmids used in this study are described in Table S1 in the supplemental material. *B. gladioli* GSRB05 and E. coli cultures were grown aerobically overnight with shaking in lysogeny broth (LB) medium (30) at 30 or 37°C, respectively. M9 medium (30) was supplemented with 0.2% glucose, 0.1 mM CaCl_2_, and 1 mM MgSO_4_. For resistance assays, antibiotics were supplemented at the following final concentrations, as indicated: 50 μg/ml streptomycin (Sm), 25 μg/ml chloramphenicol (Cm), and 100 μg/ml ampicillin (Ap).

### AST production.

The protocol for AST production was modified from the original method (8). A single colony of *B. gladioli* GSRB05 was inoculated into 10 ml of LB medium and grown overnight. The culture was 100-fold diluted into 1 liter of fresh LB and grown again to an *A*_600_ of 1. Next, the cells were harvested and transferred into the same volume of ST 10^−1^ medium (31) supplemented with 20 μM As(III) and 0.2% glucose. The culture was incubated until As(III) was completely transformed into AST. AST was chromatographically purified as described previously (8) with a yield of approximately 5 mg AST/liter of culture medium and was free of AST-OH.

The time course of As(III) conversion into AST-OH and AST by *B. gladioli* GSRB05 was performed in a 50-ml culture of ST 10^−1^ medium containing 3 μM As(III). The same procedure was used to determine As(III) transformation by E. coli Top10 bearing the vector plasmid pUC118, pUC*arsL–orf1–4*, or pUC*arsML–orf1–4* as for *B. gladioli* GSRB05, but for E. coli BL21 bearing the plasmid pETDuet-1, pETDuet-1*arsL*, pETDuet-1*arsM*, or pETDuet-1*arsLM*, the pellet was directly transferred from the overnight LB culture into ST 10^−1^ medium supplemented with 1 μM As(III) and 0.4% glycerol, following which cultures were incubated for 36 h. For both E. coli Top10 and BL21, basal expression of genes in plasmids pUC118 and pETDuet-1 was sufficient to observe the activity of the genes.

### Analysis of arsenic species.

Arsenic species in the supernatant were analyzed by high-performance liquid chromatography-inductively coupled plasma mass spectrometry (HPLC-ICP-MS). For sample preparation, 0.5 ml of culture was collected and centrifuged at 13,000 rpm for 2 min at 4°C. Supernatants were filtered with 3-kDa Amicon Ultra Centrifugal filters (MilliporeSigma, St. Louis, MO, USA) for 10 min. A mobile phase of 3 mM malonic acid, 5% methanol, and tetrabutylammonium hydroxide to reach a pH of 5.9 was used to elute a C_18_ reverse-phase column at a flow rate of 1.0 ml min^−1^.

### Construction of the draft genome sequence of *B. gladioli* GSRB05.

Genome sequencing of *B. gladioli* GSRB05 was performed using the Illumina NextSeq platform at Center for Genome Technology, University of Miami, Miller School of Medicine (Miami, FL, USA). Sequence data were comprised of 7.8 million paired-end (2 × 150) reads. Quality trimming and filtering were performed using the TrimGalore (version 0.6.4) (https://www.bioinformatics.babraham.ac.uk/projects/trim_galore/) tool to remove adapter sequences, base pairs with a quality score of <30, and reads shorter than 50 bp. Quality-trimmed reads were assembled using SPAdes (version 3.13.0) (32) and ABySS (version 2.1.5) (33) at different kmers, and optimal assembly was selected as described previously (34). Genome annotation was accomplished using the NCBI Prokaryotic Genome Annotation Pipeline (35), and predictions of *arsM* orthologous genes were performed via BLAST (36) and OrthoMCL (37) sequence analysis against the predicted proteins.

### Cloning and expression.

Genomic DNA was extracted from 3 ml of a fresh overnight culture of *B. gladioli* GSRB05 using the E.Z.N.A. Bacterial DNA kit (Omega Bio-tek, Inc., Norcross, GA, USA). The gene cluster was sequentially amplified by PCR with *Pfu* Turbo high-fidelity DNA polymerase (Agilent Technologies, Inc., Santa Clara, CA, USA), and the entire cluster or groups of genes were cloned into plasmid pUC118 between the KpnI and XbaI restriction sites. To construct pUC*arsL–orf1–4*, the cloning was carried out in two steps, while for pUC*arsML–orf1–4*, an additional step was necessary (see Fig. S1 in the supplemental material) to amplify fragments of no more than 3.2 kb to avoiding incorrect base insertion. Primers were designed with unique restriction sites to serially construct the final plasmids. The amplified products were gel purified, digested with the appropriate restriction enzymes, and inserted into vector plasmid pUC118 with the first gene in frame with the *lacZ*α gene of the vector. The *arsL* and *arsML* genes were ordered from GenScript (GenScript, Piscataway, NJ, USA) and cloned into pETDuet-1 (Millipore Sigma) and *arsQ* in pTrcHisA (Thermo Fisher Scientific, Inc., Waltham, MA, USA) to construct pETDuet-1*arsL*, pETDuet-1*arsML*, and pTrc*arsQ*, respectively. Each step of cloning was verified by sequencing the fragments. The complete list of the oligonucleotides used in this study is given in Table S1.

### Site-directed mutagenesis.

The primers for site-directed mutagenesis (see Table S2 in the supplemental material) were designed using the online QuikChange Primer Design program (https://www.agilent.com/store/primerDesignProgram.jsp). A stop codon was individually inserted into each of the five genes in plasmid pUC*arsL–orf1–4* by substitution of one nucleotide at the beginning of the sequence. Unmutated plasmid pUC*arsL–orf1–4* was removed from the reaction mixture by digesting the methylated DNA with restriction enzyme DpnI (New England Biolabs, Ipswich, MA, USA). Each mutated plasmid was transformed into competent cells of E. coli TOP10 and purified, and the presence of the mutation was verified by sequencing.

### mRNA extraction, reverse transcription and quantitative real-time PCR.

An RNeasy minikit (Qiagen, Valencia, CA, USA) was used to isolate total RNA from a 3-ml culture of *B. gladioli* GSRB05 that had been cultured with or without exposure to 3 μM As(III) in ST 10^−1^ medium for 13 h. The purity and concentrations of RNA were determined from the *A*_260_ using a Synergy H4 Hybrid microplate reader (BioTek Instruments, Inc., Winooski, VT, USA). RNA integrity was verified by electrophoresis (data not shown). Reverse transcription-PCR (RT-PCR) was performed to synthesize complementary DNA (cDNA) using a Verso cDNA synthesis kit (Thermo Fisher Scientific, Inc.) according to the manufacturer's instructions. Primer sets for real-time qPCR of target genes are listed in Table S2. One microliter of each of the purified RT-PCR products corresponding to 50 ng of total RNA was amplified in a 10-μl reaction mixture containing 0.5 μM each primer set and 5 μl of iQSYBR green supermix (Bio-Rad Laboratories, Inc., Hercules, CA, USA). Real-time qPCR assays were carried out using a Realplex2 PCR instrument (Eppendorf, Hamburg, Germany) with the following cycle steps: initial denaturation for 2 min at 94°C, followed by 40 cycles of 15 s at 94°C for denaturation, and then 30 s at 50°C for annealing, and 1 min 30 s at 72°C for extension of *arsQ*, *arsM*, and *arsL.* For fragments containing sequences from *arsM* to *orf1* (*arsM–orf1*) or from *arsL* to *orf2* (*arsL–orf2*), the reaction condition was changed to 30 s at 58°C for annealing and 3 min 10 s at 72°C for extension. All data were normalized to the amount of 16S rRNA. The threshold cycle (2^–Δ^*^CT^*) was calculated to compare the expression level of each gene.

### Assays of *arsQ* function.

To examine whether the *arsQ* gene could confer AST resistance, cells of E. coli AW3110 harboring plasmid pTrcHis2A*arsQ* were grown overnight in LB medium and washed and suspended in 0.9% NaCl. The washed cells were then inoculated into M9 medium at an *A*_600_ of 0.03 and incubated for 24 h in the presence of the indicated concentrations of AST.

To determine the function of ArsQ in AST transport, the *arsQ* gene was cloned into vector plasmid pTrcHisA. Cells of E. coli AW3110 expressing plasmid pTrcHisA*arsQ* or vector plasmid pTrcHisA only were grown to an *A*_600_ of 2 at 37°C with aeration in LB medium. The cells were harvested and suspended in buffer A (75 mM HEPES-KOH [pH 7.5], 0.15 M KCl, and 1 mM MgSO_4_) at an *A*_600_ of 4. To initiate the transport reaction, 20 μM AST was added to 1 ml of cell suspension. Portions (0.1 ml) from the cell suspension were withdrawn at the indicated times, filtered through nitrocellulose filters (0.2-μm pore diameter; EMD Millipore, Billerica, MA), and washed twice at room temperature with 5 ml of buffer A. The filters were digested with 0.3 ml of concentrated HNO_3_ (68 to 70%) overnight at room temperature. The dissolved filters were incubated for 10 min at 70°C, allowed to cool to room temperature, and diluted with HPLC-grade water (MilliporeSigma) to produce a final HNO_3_ concentration of 2%. Arsenic was quantified by ICP-MS. Standard solutions were made in the range of 1 to 50 ppb in 2% nitric acid using arsenic standards (Ultra Scientific, North Kingstown, RI, USA). Protein content was determined using a Pierce bicinchoninic acid (BCA) protein assay kit (Thermo Fisher Scientific, Inc.).

Everted membrane vesicles and transport assays were performed as described previously (38). Transport assays were performed in buffer A containing 0.25 M sucrose. The reaction mixture contained 1 mg/ml membrane proteins, 40 μM AST, and 5 mM NADH in a final volume of 0.6 ml of the same buffer. Portions (0.1 ml) were withdrawn at the indicated times, filtered through 0.2-μm-pore-size nitrocellulose filters, and washed twice with 5 ml of the same buffer. Arsenic content was determined by ICP-MS.

### AST BGC distribution.

The prevalence of AST BGC was analyzed in representative organisms. GenBank accession numbers of the following bacterial genomes are given in parentheses: *B. gladioli* GSBR05 (JAGSIB000000000) is compared with putative orthologs from Burkholderia oklahomensis (NZ_UFUH01000001), Burkholderia cepacia (NZ_CADEUO010000007), Pseudomonas aeruginosa (NZ_CACPET010000007), Pseudomonas fluorescens (NZ_LVEJ01000018), Pseudomonas amygdali (NZ_LGLI01000031), *Actibacterium* sp. (NZ_JAFEUL010000009), and *Rhodobacter* sp. (NVUP01000011). Multiple alignments of the sequences of ArsM (Fig. S3), ArsL (Fig. S4), and ArsQ (Fig. S5) orthologs were performed using T-Coffee (39) and the BoxShade version 3.21 server (https://embnet.vital-it.ch/software/BOX_form.html).

### Data availability.

The draft genome sequence for *B. gladioli* GSRB05 has been deposited in NCBI under accession no. JAGSIB000000000. Raw sequence reads have been deposited in NCBI under BioProject accession no. PRJNA722678.
