# Supplementary material for: Identification of the Biosynthetic Gene Cluster for the Organoarsenical Antibiotic Arsinothricin
Source: Microbiol Spectr. 2021 Aug 11;9(1):10.1128/spectrum.00502-21. doi: 10.1128/spectrum.00502-21 (PMC8552651; doi:10.1128/spectrum.00502-21)
Supplement: SUPPLEMENTAL FILE 1 — Supplemental material. Download SPECTRUM00502-21_Supp_1_seq10.pdf, PDF file, 0.5 MB [file spectrum00502-21_supp_1_seq10.pdf]

Submitted to Microbiology Spectrum June 9, 2021

Identification of the biosynthetic gene cluster for the organoarsenical antibiotic arsinothricin

Adriana E. Galván[1,orcid.org/0000-0002-2472-0438], Ngozi P. Paul[1,orcid.org/0000-0003-0423-195X], Jian Chen[1], Kunie Yoshinaga-Sakurai[1], Sagar M. Utturkar[2], Barry P. Rosen[1,orcid.org/0000-0002-5230-4271,] and Masafumi Yoshinaga[1*,orcid.org/0000-0002-7243-1761]

[1]*Department of Cellular Biology and Pharmacology, Florida International University, Herbert Wertheim College of Medicine, Miami, U.S.A.*

[2]*Purdue University Center for Cancer Research, Purdue University, West Lafayette, U.S.A.*

***Corresponding author**: Masafumi Yoshinaga (myoshina@fiu.edu), Department of Cellular Biology and Pharmacology, Florida International University, Herbert Wertheim College of Medicine, 11200 SW 8th Street, AHC1 419G, Miami, Florida, U. S. A. 33199, Tel: (+1) 305-348-1489, Fax: (+1) 305-348-0651

Abbreviations: AST, arsinothricin (2-amino-4-(hydroxymethylarsinoyl) butanoate); AST-OH, hydroxyarsinothricin (2-amino-4-(dihydroxyarsinoyl) butanoate; ACP, 3-amino-3-carboxypropyl; MAs(III), methylarsenite; MAs(V), methylarsenate; DMAs(III), dimethylarsenite; DMAs(V), dimethylarsenate

Keywords: Arsinothricin, organoarsenical antibiotic, biosynthetic gene cluster, *Burkholderia gladioli* GSRB05

Supplemental information

Figure S1. Diagram of the sequential cloning of the AST BGC genes. DNA fragments of no more than 3.2 kb were sequentially amplified by PCR with the indicated restriction sites. The PCR products were inserted into plasmid pUC118. The first amplified PCR fragment included *orf2-4* cloned between the *Kpn*I and *Xba*I sites. In the second step *arsL* and *orf1* were amplified and cloned between the *Kpn*I and *Pac*I sites, resulting in plasmid pUC*arsL-orf1-4*. For the construction of pUC*arsML-orf1-4* or pUC*arsQML-orf1-4*, a third fragment including *arsM* or *arsQ-arsM* genes, respectively, was cloned between the *Kpn*I and *Nhe*I sites.

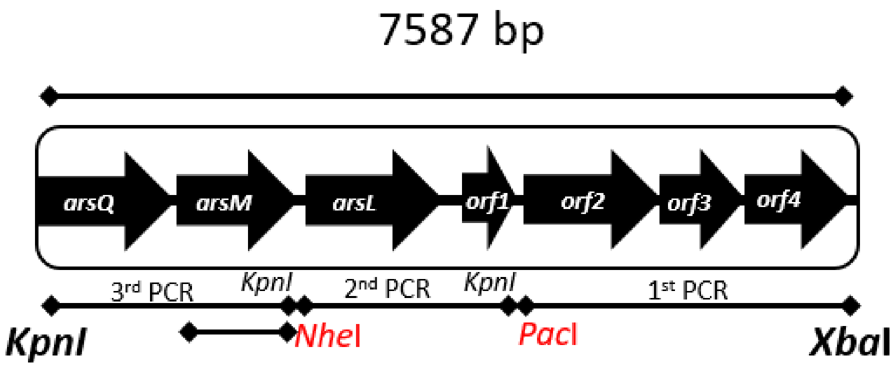

Figure S2. Genome sequencing of *B. gladioli* GSRB05

After quality trimming and filtering, >64% of reads were retained that represent >160X genome coverage for *B. gladioli* GSRB05. Optimal genome assembly obtained through SPAdes was comprised of 171 contigs in 9.2 MB with N50 contigs size 132 KB and average contig length 54 KB.

Sample_ID	file_name	Total Reads	Read Counts after quality filtering	Percentage of Reads after quality filtering
Burkholderia GSRB05	Bgladio_1.fastq	7,879,185	5,083,214	64.51%
	Bgladio_2.fastq	7,879,185	5,083,214	64.51%

Tool	KMER	Contigs	Minimum Conting Length	Maximum Conting Length	Average Contig Length	Contig N50	Assembly Size
SPAdes version 3.13.0	21,33,55,77,99,127	171	512	445,577	54,067	132,483	9,245,390
ABys version 2.1.5	31	522	507	149,544	17,564	39,307	9,168,461
	41	387	501	196,023	23,919	52,853	9,256,639
	51	364	509	198,283	25,481	53,516	9,275,193
	61	343	508	196,909	27,148	57,530	9,311,829
	71	343	505	198,297	27,150	58,494	9,312,489
	81	350	500	216,645	26,806	51,115	9,381,985
	91	408	504	150,616	22,918	45,268	9,350,690
	101	584	500	95,090	15,859	30,938	9,261,652
	111	1,316	500	62,372	6,935	14,061	9,126,425
	121	3,793	500	33,284	2,147	3,484	8,144,987

Figure S3. Multiple alignment of BgArsM orthologs (accession numbers in parentheses). The protein sequence of BgArsM identified from *B. gladioli* GSBR05 (BurGSRB05_34905) is compared with putative orthologs from *Burkholderia oklahomensis* (WP_010121994.1), *Burkholderia cepacia* (WP_059666519.1), *Pseudomonas aeruginosa* (WP_174517314.1), *Pseudomonas fluorescens* (WP_064119070.1), *Pseudomonas amygdali* (WP_054068373.1), *Actibacterium* sp. (WP_204414387.1) and *Rhodobacter* sp. (PCJ07719.1). The four conserved cysteines are indicated (*). Identities are shaded in black, and conservative replacements are shaded in grey.

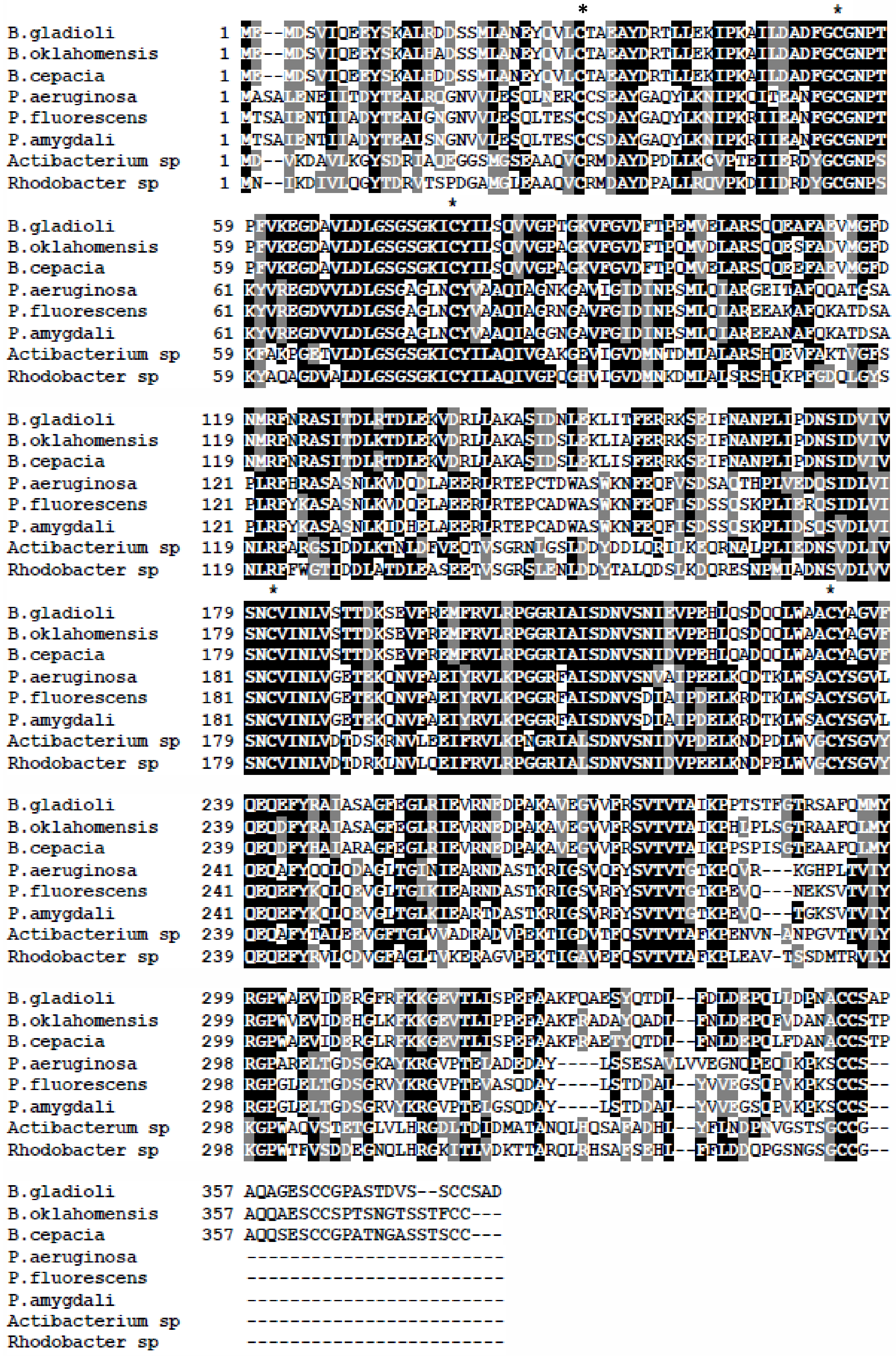

Figure S4. Multiple alignment of BgArsL orthologs (accession numbers in parentheses). The protein sequence of BgArsL identified from *B. gladioli* GSBR05 (BurGSRB05_34900) is compared with putattive orthologs from *Burkholderia oklahomensis* (WP_038802160.1), *Burkholderia cepacia* (WP_059666518.1), *Pseudomonas aeruginosa* (WP_174517312.1), *Pseudomonas fluorescens* (WP_064119068.1), *Pseudomonas amygdali* (WP_054068372.1), *Actibacterium* sp. (WP_204414396.1) and *Rhodobacter* sp. (PCJ07718.1). Identities are shaded in black, and conservative replacements are shaded in grey. The cysteines of the conserved CX_3CX_2C motif are identified (*).

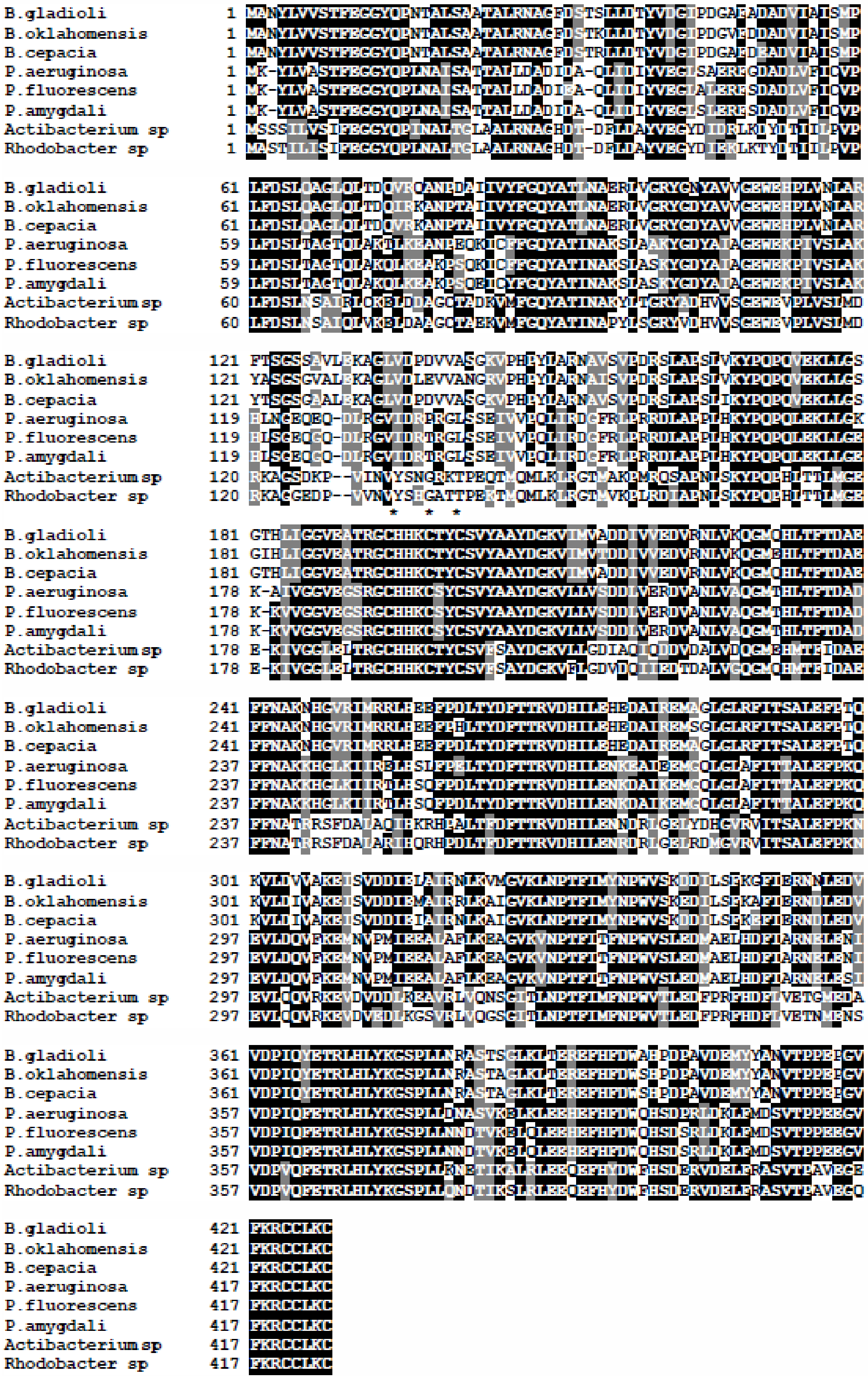

Figure S5. Multiple alignment of BgArsQ orthologs (accession numbers in parentheses). The protein sequence of BgArsQ identified from *B. gladioli* GSBR05 (BurGSRB05_34910) is compared with putative orthologs from *Burkholderia oklahomensis* (WP_010121992.1), *Burkholderia cepacia* (WP_081062335.1), *Pseudomonas aeruginosa* (WP_174517311.1), *Pseudomonas fluorescens* (WP_064119067.1), *Pseudomonas amygdali* (WP_054068371.1), *Actibacterium* sp. (WP_204414385.1) and *Rhodobacter* sp. (PCJ07720.1). Identities are shaded in black, and conservative replacements are shaded in grey.

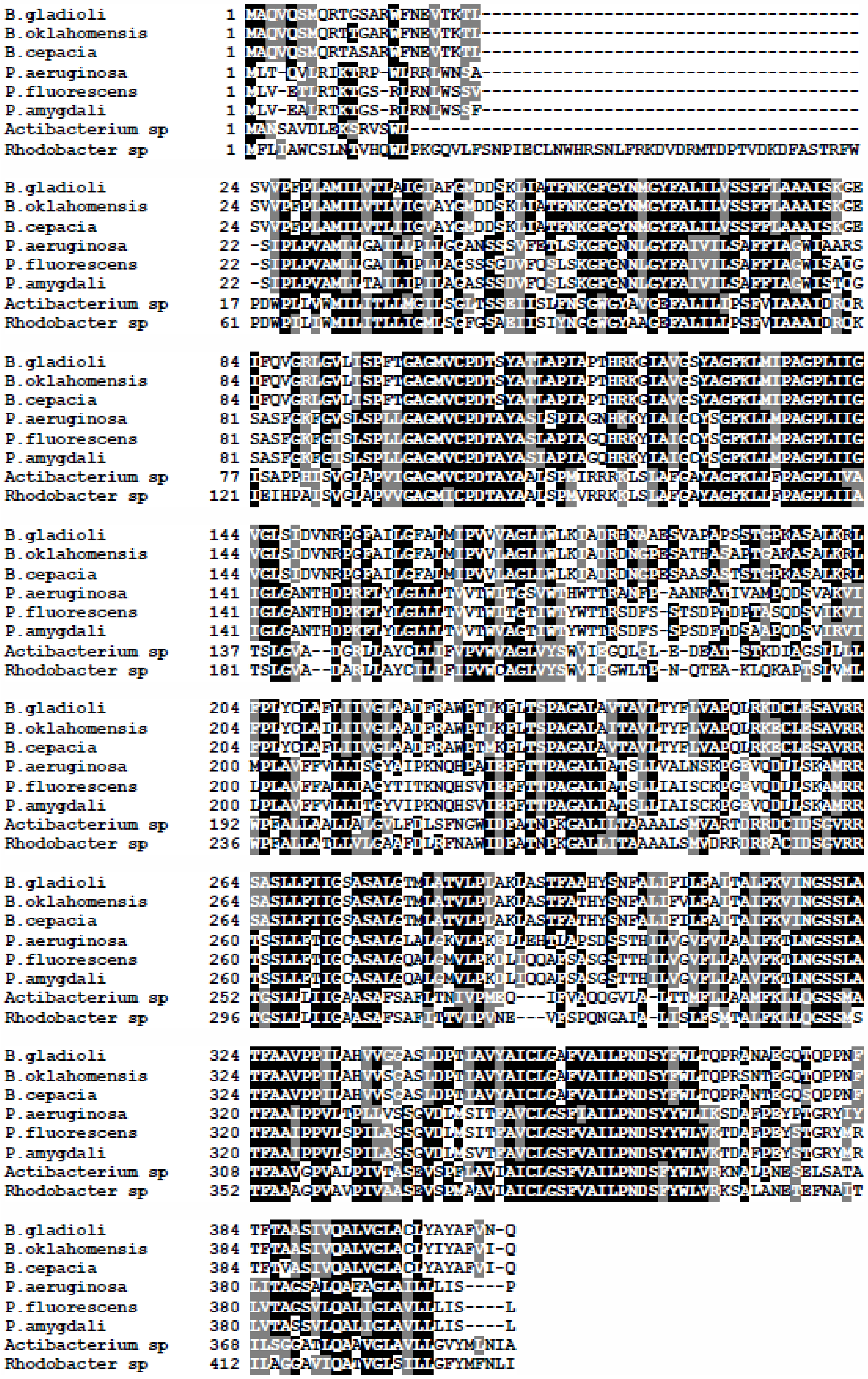

Table S1. Bacterial strains and plasmids

	Genotype/Description	References
Strain		
B. gladioli GSRB05	Soil isolated. AST producer	
E. coli Top10	F⁻ *mcr*A Δ(*mrr-hsd*RMS-*mcr*BC) φ80*lac*ZΔM15 Δ*lac*X74 *rec*A1 *ara*D139 Δ(*ara-leu*)7697 *gal*U *gal*K λ⁻ *rps*L(Str^{R}) *end*A1 *nup*G. Sm^{r}	Invitrogen
E. coli BL21 (DE3)	F⁻ *omp*T *hsd*S_B (r_B^-, m_B^-) *gal dcm* (DE3)	Novagen
E. coli AW3110	*ars*::*cam* F- IN(*rrnD-rrnE*). Cm^{r}	(1)
Plasmid		
pUC118	*E. coli* cloning and expression using *lac* promoter. Ap^{r}	Takara Bio USA
pUC*arsL-orf1-4*	PCR amplified *arsL* and orf1-4 genes inserted into the *Kpn*I and *Xba*I sites of pUC118.	This study
pUC*arsML-orf1-4*	PCR amplified *arsM-arsL* and orf1-4 genes inserted into the *Kpn*I and *Xba*I sites of pUC118	This study
pUC*arsQML-orf1-4*	PCR amplified *arsQ-arsM-arsL* and orf1-4 genes inserted into the *Kpn*I and *Xba*I sites of pUC118	This study
pETDuet-1	*E. coli* expression vector. Ap^{r}	Novagen
pETDuet-1*arsL*	*arsM* gene cloned in MCSI	This study
pETDuet1*arsML*	*arsM* gene cloned in MCSI and *arsL* gene cloned in MCSII	This study
pTrcHisA	*E. coli* expression vector. Ap^{r}	Thermo Fisher Scientific Inc
pTrcHisA2*arsQ*	*arsQ* genes inserted into the *Nco*I and *Sal*I sites of pTrcHisA.	This study

Abbreviations: Sm^{r}, streptomycin resistant. Cm^{r}, chloramphenicol resistant. Ap^{r}, ampicillin resistant.

1. Carlin, A.; Shi, W.; Dey, S.; Rosen, B. P., The *ars* operon of *Escherichia coli* confers arsenical and antimonial resistance. J. Bacteriol. 1995, 177, (4), 981-6.

Table S2. Primers

Cloning rimers	Sequence
Bg_ars_XbaI_Rv	5’ ACCACCACCTCTAGATGGGGCATAGCGATAG 3’
Bg_ars_KpnI PacI_Fw	5’ GGTGGTGGTACCTTAATTAAGTGATAGCACGTCCGTCATTG 3’
Bg_ars_PacI_Rv	5’ ACCACCTTAATTAAGTGGCGCATGCCGGAGG 3’
Bg_ars_KpnI_NheI _Fw	5’ GGTGGTGGTACCGCTAGCTGAATAGATCAACAGACGACTTCAAG 3’
Bg_ars_NheI_Rv	5’ ACCACCGCTAGCATCAGCCGAGCAGCAG 3’
Bg_arsL_KpnI_Fw	5’ GGTGGTGGTACCTGGCCCAGGTTCAATC 3’
Bg_arsM_KpnI_Fw	5’ GGTGGTGGTACCAATGGAAATGGATTCTGTTATTC 3’

Red: unique site introduced for the sequentially cloning

Site-mutant primers	
arsL-G31T-Fwd	5’ CTGATATCCACCTTAAAAAGTGGAAACGACTAGATAGTTGG 3’
arsL-G31T-Rev	5’ CCAACTATCTAGTCGTTTCCACTTTTTAAGGTGGATATCAG 3’
orf1-C55T-Fwd	5’ CATCGAGCATTTCCTACACGGGGTCTATCGC 3’
orf1-C55T-Rev	5’ GCGATAGACCCCGTGTAGGAAATGCTCGATG 3’
orf2 -G28T-Fwd	5’ GCGACCGGTTATTCCTTGGCGAGTGCTGC 3’
orf2 -G28T-Rev	5’ GCAGCACTCGCCAAGGAATAACCGGTCGC 3’
orf3-G31T-Fwd	5’ CGGTATAGAAGAGCTAAACCCCGTCGTTAGTTCT 3’
orf3-G31T-Rev	5’ AGAACTAACGACGGGGTTTAGCTCTTCTATACCG 3’
orf4-C28T-Fwd	5’ CGCGTCGGGCTAGGCGCTGCTGA 3’
orf4-C28T-Rev	5’ TCAGCAGCGCCTAGCCCGACGCG 3’

Primers used in *ars* operon identification by RT-qPCR	
1F	5’ ATGGCCCAGGTTCAATCGATG 3’
1R	5’ CCTGAATAACAGAATCCATTTCC 3’
2F	5’ ATGGAAATGGATTCTGTTATTCAG 3’
2R	5’ GTGGAAACGACTAGATAGTTG 3’
3F	5’ ATGGCCAACTATCTAGTCGTTTC 3’
3R	5’ TCAGCATTTGAGGCAGCATCG 3’
4R	5’ GAAAACGCCGATGCCGTAC 3’
5R	5’ AGAGTGAAATCGTCATCGATTG 3’
27F-16S	5’ AGAGTTTGATCTGGCTCAG 3’
1492R-16S	5’ GGTTACCTTGTTACGACTT 3’
